# Supplementary material for: Real-Life Considerations on Antifungal Treatment Combinations for the Management of Invasive Mold Infections after Allogeneic Cell Transplantation
Source: J Fungi (Basel). 2021 Sep 28;7(10):811. doi: 10.3390/jof7100811 (PMC8540382; doi:10.3390/jof7100811)
Supplement: Supplementary file 1 [file jof-07-00811-s001.zip › jof-1382101-supplementary.pdf]

Supplementary material

Figure legends

Supplementary Figure S1. Kaplan-Meier survival curves of all-cause-1-year mortality after the diagnosis of an invasive mold infection (IMI) according to IMI type: invasive aspergillosis (IA) versus non-IA IMI.

Supplementary Figure S1.

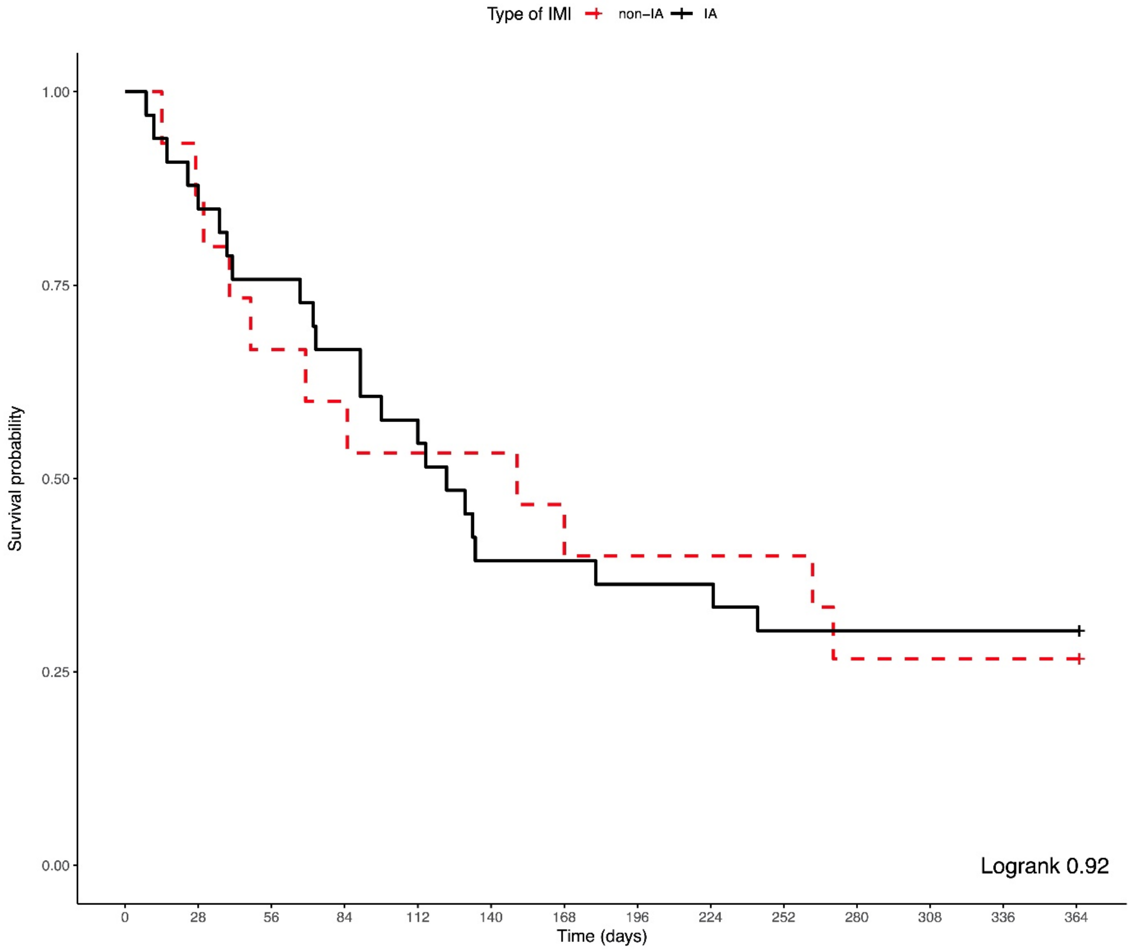

Number at risk

	0	28	56	84	112	140	168	196	224	252	280	308	336	364
non−IA	15	13	10	9	8	8	7	6	6	6	4	4	4	4
IA	33	29	25	22	19	13	13	12	12	10	10	10	10	10
